# Impact of medication adherence on renal function in comorbid patients with type 2 diabetes and depression: protocol for a cohort study

**DOI:** 10.1186/s12875-015-0339-1

**Published:** 2015-09-15

**Authors:** Hiroto Ito, Tosiya Sato, Noriko Satoh-Asahara, Mitsuhiko Noda

**Affiliations:** Department of Social Psychiatry, National Institute of Mental Health, National Center of Neurology and Psychiatry, Tokyo, Japan; Department of Biostatistics, Kyoto University School of Public Health, Kyoto, Japan; Division of Diabetic Research, Clinical Research Institute, National Hospital Organization Kyoto Medical Center, Kyoto, Japan; Department of Diabetes and Metabolic Medicine, National Centre for Global Health and Medicine, Tokyo, Japan

**Keywords:** Chronic kidney disease, Depression, Medication adherence, Protocol, Type 2 diabetes

## Abstract

**Background:**

Prevention, or slowing the progression, of diabetic nephropathy is one of the important goals in diabetes care. Although the impact of depression is a concern in patients with diabetes, it is unknown whether there is an association between adherence to hypoglycemic medication and the decline of renal function in comorbid patients with diabetes and depression. We will conduct a cohort study aimed at examining (1) depression as a predictor of renal function decline, and (2) how adherence to hypoglycemic medication relates to depression and renal function decline in patients with type 2 diabetes.

**Methods/Design:**

In this multicenter cohort study, 550 patients with type 2 diabetes aged 20 years and older will be recruited at 20 outpatient clinics in general medicine and psychiatry. We will measure depression (Patient Health Questionnaire), medication adherence (medication possession ratio, Morisky Medication Adherence Scale, and one-item hypoglycemic medication adherence scale), and renal function (urinary albumin-creatinine ratio and estimated glomerular filtration rate) at baseline and at the 12-month follow-up. The primary endpoint is decline of renal function at 12 months. The secondary endpoints include clinical variables, quality of life, and the attitude of professionals toward depression. We will perform multivariable linear regression analysis to evaluate the effects of medication adherence on the decline of renal function in comorbid patients with type 2 diabetes and depression.

**Discussion:**

To our knowledge, this will be the first study to examine how adherence to hypoglycemic medication relates to the decline of renal function in comorbid patients with type 2 diabetes and depression. The results of the study will have implications for practitioners of diabetes care, policy makers, and researchers for the prevention and treatment of diabetic nephropathy.

**Trial registration:**

UMIN000017513 (Registered on May 22, 2015)

## Background

Prevention, or slowing the progression, of diabetic nephropathy is one of the important goals in diabetes care [1, 2]. As the kidney disease progresses, end-stage renal disease (ESRD) occurs and requires either dialysis or transplantation, which places significant burden on the patient and the society. Diabetes is one of the predictors of a poor perceived quality of life in patients with ESRD [3], and the prevalence of diabetes could impose an increasing economic burden due to substantial costs for patients with ESRD [4].

Depression is observed in approximately 20 % of patients with diabetes [5, 6]. Although adherence to hypoglycemic medication is important for the prevention and management of diabetic nephropathy [2], a meta-analysis indicates that depression is associated with lower adherence to hypoglycemic medication in patients with diabetes [7]. Furthermore, depression also adversely affects adherence to other key medications for hypertension and hyperlipidemia - the main risk factors of diabetic nephropathy [8]. Considering the high prevalence of depression and that depression is rather commonly undiagnosed in patients with diabetes [9], the impact of depression on diabetic nephropathy is a growing concern.

The literature has reported the morbidity and mortality of kidney disease in patients with depression. A recent meta-analysis showed that depression was observed in 21.4 % of patients in chronic kidney disease (CKD) stage 1–5 [10]. Comorbid patients with diabetes and depression had higher risks of microalbuminuria [11] and ESRD than those without depression [12]. Patients with diabetic nephropathy who have had a depressive episode were more likely undergo dialysis than those without a depressive episode [13], and the mortality was 1.5-fold higher in diabetes patients with depression than in those without depression [14, 15].

In patients with type 2 diabetes, we aim to examine (1) depression as a predictor of renal function decline, and (2) how adherence to hypoglycemic medication relates to depression and renal function decline.

## Methods/design

### Study design

This is a multicenter cohort study. We refer STROBE reporting guidelines to this protocol (http://www.strobe-statement.org/).

### Ethical approval

The study protocol was approved by the institutional review board (IRB) of the National Center of Neurology and Psychiatry (NCNP) and participating clinics with IRBs. The IRB of the NCNP approved the protocol for those without IRB. This study is in accordance with the Ethical Guidelines for Epidemiological Research (http://www.niph.go.jp/wadai/ekigakurinri/guidelines.pdf).

### Study setting

This multicenter study will be conducted at 20 outpatient clinics (NCNP, 5 from the Japan Diabetes Mellitus Effective Network System, and 14 from the Stress-Care Inpatient Care Network) in Japan. The Japan Diabetes Mellitus Effective Network System is a network of primary care physicians and specialists of diabetes collaborating to prevent diabetes complications, whereas the Stress-Care Inpatient Care Network consists of leading psychiatrists aiming to improve the quality of psychiatric care for patients with depression.

### Participants

All patients who meet inclusion criteria during the study period will be invited to participate in the study. Participants will be eligible for the study if they (1) are 20 years and older, (2) outpatients with type 2 diabetes receiving hypoglycemic medications, and (3) can complete a questionnaire in Japanese. Patients will be excluded if they (1) have cognitive impairment, (2) are pregnant, and (3) have severe disease of the adrenal glands, liver dysfunction, or severe renal impairment (serum creatinine ≥ 2.0 mg/dL).

Eligible patients will be approached by physicians at each site. After an explanation of the study, written informed consent will be obtained from each patient. Patients will be able to access research staff members who are not in charge of their treatment to withdraw from the study at any time.

### Study endpoints

The primary outcome is the decline of renal function in four subtypes categorized according to depression and adherence (depression/adherence: +/−, +/+, −/+, −/−) at 12 months. The secondary outcomes include clinical variables, quality of life, and attitude of physicians toward depression. We assess physician attitudes to depression to confirm no effects of the attitudes on primary outcome in a sensitivity analysis. Previous systematic review suggests necessity for cohort studies to examine information on patients with multimorbidity across all ages and socioeconomic groups [16]. Although analyses of secondary outcomes will be underpowered, we include the analyses in the protocol for confirmation of the analysis of primary outcome and implication for the future studies.

### Data collection

Data will be collected at routine clinic visits by coordinating staff at baseline and 12 months. The data include patients’ demographic variables (e.g., age, gender, and smoking status), body mass index, prescription, blood pressure, laboratory data (e.g., glucose level, HbA1c, total cholesterol, low- or high-density lipoprotein cholesterol, triglycerides, serum creatinine, and urinary albumin-creatinine ratio [ACR]), and diabetic complications. Table 1 shows the primary measures and timings for measurement.

**Table 1:** Measures and timings for measurements

	Scale	Baseline	1 Year
Renal function	urinary albumin-creatinine ratio (ACR) estimated glomerular filtration rate (eGFR)	●	●
Depression	Patient Health Questionnaire (9-item): PHQ-9	●	●
Adherence	Medication Possession Ratio (MPR) Morisky Medication Adherence Scale (MMAS-8) Oral Hypoglycemic Agent (OHA) adherence	●	●
Quality of life	EuroQol	●	●

### Renal function

Renal function will be assessed on the basis of ACR and estimated glomerular filtration rate (eGFR) [17, 18], and the decline of renal function is defined as ACR ≥ 30 mg/g creatinine and eGFR < 60 mL⋅min^−1^⋅1.73 m^−2^.

### Depression

Depression will be assessed by using the Patient Health Questionnaire-9 (PHQ-9). The PHQ-9 is widely used in patients with chronic diseases, including diabetes [8]. On the basis of a previous study [11], we operationally define participants with PHQ-9 scores ≥ 10 as having depressive symptoms. The reliability and validity of the Japanese version of PHQ-9 have been established [19]. In this study, we define depression as having clinical diagnosis (DSM-5, American Psychiatric Association) [20] or having both severe depressive symptoms and receiving antidepressant.

### Medication adherence

Medication adherence will be assessed by using the medication possession ratio (MPR). The MPR is defined as the ratio of the number of days prescribed per 365 days based on medical records. The MPR is used as an indicator for adherence improvement programs [21, 22]. In this study, we operationally define low adherence as < 80 % because a previous study reported that the mean adherence ranged between 78 % and 82 % [23]. Medication adherence will also be assessed by using the Japanese version of the Morisky Medication Adherence Scores (MMAS-8). MMAS-8 is an eight-item self-reporting measure of medication-taking behavior [24, 25]. The first seven items have a dichotomous response (yes or no); the eighth item has a five-point Likert scale response. The total score of MMAS-8 ranges from 0 to 8. A higher score represents a higher adherence to medication. In addition, a single-question Oral Hypoglycemic Agent (OHA) adherence scale will be used specifically to assess adherence to hypoglycemic medication [23]. The question is, “On average, how would you rate your ability to take all your diabetes medications as your doctor prescribed?,” answered with a five-point Likert scale (very poor, poor, fair, good, very good, and excellent).

### Quality of life

The quality of life of patients will be assessed by using EuroQol. EuroQol is a generic measure of the respondents’ health-related quality of life and utility values [26, 27]. The questionnaire consists of five items covering the areas of mobility, self-care, usual activities, pain/discomfort, and anxiety/depression (EQ-5D). Each item has three levels: no problems, some problems, or extreme problems. The EQ-5D scores are converted to a single index value (ranging from 0 for the worst health state to 1 for the best health state) [27]. A higher score represents a higher quality of life. The reliability and validity of the Japanese version have been established [28], and used in patients with diabetes in Japan [29].

### Attitude of physicians toward depression

Attitudes of physicians toward depression will be measured by using the Depression Attitude Questionnaire (DAQ) [30]. This questionnaire consists of 20 items based on three themes: the conceptual model of the responder, the respondents’ value judgments, and their practical response. Answers are marked on a 100-mm visual analogue scale ranging from “strongly disagree” (0 mm) to “strongly agree” (100 mm). The DAQ has been translated into Japanese, and its reliability and validity have been verified [31]. Receiving completed DAQ will be regarded as the physicians agree to participate in the questionnaire survey.

### Sample-size calculations

We calculated sample size as 550 patients (165 for the depression group and 385 for the non-depression group) having (1) a 30 % prevalence of depression, (2) 10 % difference in microalbuminuria rate (18 % in the depression group and 8 % in the non-depression group), and (3) an 88 % chance of detecting a true difference in microalbuminuria at the 5 % level of significance. Calculation was based on the chi-square test with continuity correction and we did not take into account of adherence since it might be an intermediate variable. The prevalence of depression in patients with diabetes was 20 % in previous studies [5, 6, 10, 11]; however, we estimated 30 % owing to the inclusion of patients from psychiatric clinics in our study. The prevalence of microalbuminuria was estimated by using the data of the Japanese Society of Nephrology [18] and those from a previous study [11].

### Data analysis

We will use multivariable linear regression analysis to evaluate the effects of medication adherence of comorbid patients with type 2 diabetes and depression on the decline of renal function. We will consider other potential confounding factors including smoking, hypertension, hyperlipidemia, and HbA1c. The risk of decline of renal function will be estimated for four groups by adherence (+/−) and depression (+/−) by odds ratio and 95 % confidence interval.

## Discussion

To our knowledge, the present study will be the first study to examine how adherence to hypoglycemic medication relates to the decline of renal function in comorbid patients with type 2 diabetes and depression. Given the prevalence of diabetes and depression, the impact of the comorbidity of these diseases is increasingly important. We will use all standardized measures. Renal function is assessed by urinary ACR and eGFR. As an increase of ACR is observed earlier than a decrease of eGFR in CKD, our study design aids in the better understanding of renal function decline from the early phase in comorbid patients with type 2 diabetes and depression. Adherence is assessed by both objective (MPR) and subjective (MMAS-8 and OHA) measures. MMAS-8 measures general adherence to medication, whereas OHA specifically measures adherence to hypoglycemic medication. A wide range of outcomes, including clinical variables, quality of life, and attitude of professionals toward depression, are also assessed.

This study design has several limitations. First, the participants will be patients who regularly visit clinic, causing a risk of bias. This might limit the generalizability of the findings. Second, our study will not include all possible confounding factors related to diabetes, depression, hypoglycemic medication adherence, and decline of renal function; nevertheless, we plan to control the major potential confounding factors.

The results of the present study will have implications to practitioners in diabetes care, policy makers, and researchers for screening a group of patients at a high risk for diabetic nephropathy (i.e., comorbid patients with type 2 diabetes and depression plus poor adherence to hypoglycemic medication) and for the development of intervention strategies for the prevention and treatment of diabetic nephropathy.

In conclusion, the results of this protocol study will indicate the importance of depression in diabetic care through exploring (1) depression as a predictor of renal function decline, and (2) how adherence to hypoglycemic medication relates to depression and renal function decline. Our research will provide insights to all clinicians who encounter comorbid patients with diabetes and depression in dairy practice.

## Abbreviations

ACR: Albumin-creatinine ratio
CKD: Chronic kidney disease
DAQ: Depression attitude questionnaire
eGFR: Estimated glomerular filtration rate
ESRD: End-stage renal disease
IRB: Institutional review board
MMAS-8: Morisky medication adherence scale-8
MPR: Medication possession ratio
NCNP: National Center of Neurology and Psychiatry
OHA: Oral hypoglycemic agent
PHQ-9: Patient health questionnaire-9

## References

[1] Gross JL, de Azevedo MJ, Silveiro SP, Canani LH, Caramori ML, Zelmanovitz T (2005). Diabetic nephropathy: diagnosis, prevention, and treatment. Diabetes Care.

[2] Hall PM (2006). Prevention of Progression in Diabetic Nephropathy. Diabetes Spectrum.

[3] Moura A, Madureira J, Alija P, Fernandes JC, Oliveira JG, Lopez M, et al. Predictors of health-related quality of life perceived by end-stage renal disease patients under online hemodiafiltration. Qual Life Res. DOI 10.1007/s11136-014-0854-x [Epub ahead of print]. 10.1007/s11136-014-0854-x 25381124

[4] Joyce AT, Iacoviello JM, Nag S, Sajjan S, Jilinskaia E, Throop D (2004). End-stage renal disease-associated managed care costs among patients with and without diabetes. Diabetes Care.

[5] Ali S, Stone MA, Peters JL, Davies MJ, Khunti K (2006). The prevalence of co-morbid depression in adults with Type 2 diabetes: a systematic review and meta-analysis. Diabet Med.

[6] Barnard KD, Skinner TC, Peveler R (2006). The prevalence of co-morbid depression in adults with Type 1 diabetes: systematic literature review. Diabet Med.

[7] Gonzalez JS, Peyrot M, McCarl LA, Collins EM, Serpa L, Mimiaga MJ (2008). Depression and diabetes treatment nonadherence: a meta-analysis. Diabetes Care.

[8] Lin EH, Katon W, Von Korff M, Rutter C, Simon GE, Oliver M (2004). Relationship of depression and diabetes self-care, medication adherence, and preventive care. Diabetes Care.

[9] Li C, Ford ES, Zhao G, Ahluwalia IB, Pearson WS, Mokdad AH (2009). Prevalence and correlates of undiagnosed depression among U.S. adults with diabetes: the Behavioral Risk Factor Surveillance System, 2006. Diabetes Res Clin Pract.

[10] Palmer S, Vecchio M, Craig JC, Tonelli M, Johnson DW, Nicolucci A (2013). Prevalence of depression in chronic kidney disease: systematic review and meta-analysis of observational studies. Kidney Int.

[11] Yu MK, Katon WA, Young BA (2013). Diabetes self-care, major depression, and chronic kidney disease in an outpatient diabetic population. Nephron Clin Pract.

[12] Yu MK, Weiss NS, Ding X, Katon WJ, Zhou XH, Young BA (2014). Associations between Depressive Symptoms and Incident ESRD in a Diabetic Cohort. Clin J Am Soc Nephrol.

[13] Hedayati SS, Minhajuddin AT, Afshar M, Toto RD, Trivedi MH, Rush AJ (2010). Association between major depressive episodes in patients with chronic kidney disease and initiation of dialysis, hospitalization, or death. JAMA.

[14] Farrokhi F, Abedi N, Beyene J, Kurdyak P, Jassal SV (2014). Association between depression and mortality in patients receiving long-term dialysis: a systematic review and meta-analysis. Am J Kidney Dis.

[15] Palmer SC, Vecchio M, Craig JC, Tonelli M, Johnson DW, Nicolucci A (2013). Association between depression and death in people with CKD: a meta-analysis of cohort studies. Am J Kidney Dis.

[16] Smith SM, Soubhi H, Fortin M, Hudon C, O’Dowd T (2012). Managing patients with multimorbidity: systematic review of interventions in primary care and community settings. BMJ.

[17] International Society of Nephrology (2013). KDIGO 2012 Clinical Practice Guideline for the Evaluation and Management of Chronic Kidney Disease Assessments. Kidney Int.

[18] Japanese Society of Nephrology (2012). Clinical Practice Guidebook for Diagnosis and Treatment of Chronic Kidney Disease 2012.

[19] Muramatsu K, Miyaoka H, Kamijima K, Muramatsu Y, Yoshida M, Otsubo T (2007). The patient health questionnaire, Japanese version: validity according to the mini-international neuropsychiatric interview-plus. Psychol Rep.

[20] American Psychiatric Association (2013). Diagnostic and statistical manual of mental disorders (DSM-5).

[21] Steiner JF, Prochazka AV (1997). The assessment of refill compliance using pharmacy records: methods, validity, and applications. J Clin Epidemiol.

[22] Vink NM, Klungel OH, Stolk RP, Denig P (2009). Comparison of various measures for assessing medication refill adherence using prescription data. Pharmacoepidemiol Drug Saf.

[23] Gonzalez JS, Schneider HE, Wexler DJ, Psaros C, Delahanty LM, Cagliero E (2013). Validity of medication adherence self-reports in adults with type 2 diabetes. Diabetes Care.

[24] Morisky DE, Ang A, Krousel-Wood M, Ward H (2008). Predictive Validity of a Medication Adherence Measure for Hypertension Control. J Clin Hypertens.

[25] Morisky DE, DiMatteo MR (2011). Improving the measurement of self-reported medication nonadherence: Final response. J Clin Epidemio.

[26] Group EQ (1990). EuroQol: a new facility for the measurement of health related quality of life. Health Policy.

[27] Rabin R, de Charro F (2001). EQ-5D: A measure of health status from the EuroQol Group. Ann Med.

[28] Japanese EuroQol Translation Team (1998). The development of the Japanese EuroQol instrument. Medicine and Society.

[29] Sakamaki H, Ikeda S, Ikegami N, Uchigata Y, Iwamoto Y, Origasa H (2006). Measurement of HRQL using EQ-5D in patients with type 2 diabetes mellitus in Japan. Value Health.

[30] Botega N, Mann A, Blizard R, Wilkinson G (1992). General practitioners and depression-first use of the depression attitude questionnaire. Int J Methods Psychiatr Res.

[31] Ohtsuki T, Kodaka M, Sakai R, Ishikura F, Watanabe Y, Mann A (2012). Attitudes toward depression among Japanese non-psychiatric medical doctors: a cross-sectional study. BMC Res Notes.

