# Erratum to: Impact of medication adherence on renal function in comorbid patients with type 2 diabetes and depression: protocol for a cohort study

**DOI:** 10.1186/s12875-017-0629-x

**Published:** 2017-05-10

**Authors:** Hiroto Ito, Tosiya Sato, Noriko Satoh-Asahara, Mitsuhiko Noda

**Affiliations:** 1 Department of Social Psychiatry, National Institute of Mental Health, National Center of Neurology and Psychiatry, Tokyo, Japan; 2 Department of Biostatistics, Kyoto University School of Public Health, Kyoto, Japan; 3 Division of Diabetic Research, Clinical Research Institute, National Hospital Organization Kyoto Medical Center, Kyoto, Japan; 4 Department of Diabetes and Metabolic Medicine, National Centre for Global Health and Medicine, Tokyo, Japan

## Erratum

The authors of this study protocol [1] would like to amend it and clarify that one of the medication adherence scales, the Morisky Medication Adherence Scale 8 (MMAS-8) [2–4], will not be used. This amendment will not cause any changes to the substantive contents of the article since the medication adherence will be assessed by the use of other measures. The Institutional Review Board of the National Center of Neurology and Psychiatry approved this change to the study protocol.
